# Association Between Injury Etiology and Scar Outcomes: A Retrospective Clinical Study

**DOI:** 10.1111/jocd.71042

**Published:** 2026-07-09

**Authors:** Runzhi Huang, Leibing Zhang, Mingkun Ma, Dayuan Xu, Hongda Bi, Shizhao Ji

**Affiliations:** ^1^ Department of Burn Surgery The First Affiliated Hospital of Naval Medical University Shanghai China; ^2^ Department of Plastic Surgery The First Affiliated Hospital of Naval Medical University Shanghai China

**Keywords:** burn injury, injury etiology, predictive model, risk stratification, scar prognosis, wound healing

## Abstract

**Background:**

While scar formation significantly impacts the quality of life post‐injury, the prognostic value of the initial injury etiology remains uncertain. This study aimed to elucidate the relationship between injury etiology and scar prognosis, and to develop a clinically applicable predictive tool and clinical treatment strategies.

**Methods:**

In this single‐center retrospective cohort study, clinical data pertaining to 1 160 scar patients who completed a 1.5‐year follow‐up were analyzed at Changhai Hospital from 2013 to 2023. Demographic, injury etiology, treatment, and outcome data were collected for subsequent analysis. A prognostic nomogram was developed based on significant predictors identified through univariate and multivariate logistic regression. Its performance was validated using receiver operating characteristic (ROC) curves, calibration plots, and decision curve analysis (DCA). Model interpretability was enhanced using SHapley Additive exPlanations (SHAP). Subgroup analyses explored mediating factors.

**Results:**

Injury etiology was a significant predictor of scar prognosis (*p* < 0.001). Burn injury was associated with the highest risk of non‐improvement (OR = 2.36, 95% CI: 1.62–3.44). The nomogram, incorporating gender, age, injury etiology, and scar stage at initial treatment, showed modest discriminatory performance (AUC: 0.606 in training, 0.633 in test sets). SHAP analysis confirmed injury etiology as the influential predictive feature. Subgroup analyses revealed that burn wounds were characterized by a larger area, greater depth, prolonged healing time, and subsequently, a longer scar formation latency.

**Conclusion:**

The study found that injury etiology was significantly correlated with scar prognosis, and burn‐induced scars presented with significantly poorer prognosis than other etiologies. This might be attributed to the depth of tissue damage, a sustained pro‐fibrotic inflammatory response, and prolonged wound healing time commonly observed in burn injuries. It can be inferred that early identification of high‐risk patients based on injury etiology is crucial for initiating timely and intensive scar management to improve outcomes.

## 1 Introduction

Pathological scarring, which mainly encompasses hypertrophic scars and keloids, arises from aberrant tissue repair and excessive fibrotic responses following dermal injury. Beyond cosmetic disfigurement and functional impairment, pathological scarring is frequently accompanied by persistent pain and pruritus, leading to substantial physical and psychological burden [1, 2, 3]. Despite advances in understanding the molecular mechanisms of scar formation, predicting scar progression and tailoring preventive or therapeutic strategies remain challenging in clinical practice.

Scar formation is a complex biological process involving inflammation, angiogenesis, and extracellular matrix remodeling, regulated by multiple factors including ethnicity, age, genetic background, infection, and injury etiology [4]. Among these factors, injury etiology, as an upstream determinant of wound healing, is often underestimated. Existing research has predominantly focused on scar outcomes and remodeling, often overlooking how injury etiology shapes wound healing dynamics [5]. The nature of the inciting injury, whether damage from burns, trauma, or surgery, plays a pivotal role in shaping the inflammatory milieu, cellular responses, and matrix remodeling that ultimately determine scar quality. For instance, burns often induce extensive tissue necrosis and a robust, prolonged inflammatory response, creating a microenvironment that predisposes to hypertrophic scarring [6, 7]. In contrast, trauma may involve variable tissue loss and contamination, whereas surgical wounds, typically controlled and clean, follow a more predictable healing course but remain influenced by tissue tension and host factors [8]. Previous studies have largely focused on single injury mechanisms or specific treatment modalities [9, 10], with few large clinical studies directly comparing scar characteristics and prognosis across different injury etiologies. In particular, systematic quantitative analyses comparing objective scar features, such as color, thickness, vascularity, and pliability, across burns, trauma, and surgical incisions are lacking. Although the Vancouver Scar Scale (VSS) is regarded as one of the gold standards for clinical scar assessment, there remains a lack of effective models in clinical practice that can integrate injury etiology and patients' baseline characteristics to early predict the response to scar treatment [11]. Clarifying the significant predictive value of different injury etiologies for prognosis is of great significance for guiding clinicians in formulating stratified treatment strategies.

Based on the clinical data of 1 160 scar patients treated at Changhai Hospital between 2013 and 2023, this study systematically analyzed the correlations between different injury etiologies and various VSS parameters, as well as patients' subjective symptoms (pain, pruritus), with a follow‐up period of 1.5 years. The aim of this study is to construct a visual nomogram prediction model through multivariate logistic regression, reveal the significant predictive role of injury etiology in scar prognosis, and explore their inherent associations with demographic characteristics, injury severity, and treatment adherence. This study intends to provide evidence‐based medical evidence for the clinical development of precise scar prevention and treatment strategies.

## 2 Materials and Methods

### 2.1 Data Source

This single‐center retrospective cohort study was approved by the Ethics Committee of Changhai Hospital (approval number: CHEC2024‐410). A total of 1 160 patients who received scar treatment at the hospital between January 2013 and December 2023 were enrolled, with a 1.5‐year follow‐up. Injury etiology was categorized as burns, trauma, or surgery, which constituted the primary exposure groups of interest. Other injury etiology, including acne‐related scars and post‐inflammatory sequelae, was classified as “others”, which accounted for a relatively low proportion in the study cohort and thus was not the focus of the primary analyses.

Scar severity was assessed using the VSS, which evaluates pigmentation (M), height (H), vascularity (V), and pliability (P). Additionally, pain intensity was assessed using the Visual Analogue Scale (VAS). Pruritus severity and objectively measured scar thickness (OMST) were recorded at the same visits. The primary outcome was improvement based on changes in the total VSS score. Significant improvement was defined as a reduction of at least 20% in the total VSS score from baseline at the end of treatment. Patients who did not meet this criterion were classified as having non‐significant improvement. Secondary outcome measures included improvements in the four individual VSS subscales (M, H, V, P), changes in VAS scores, alterations in OMST, and the degree of pruritus relief.

### 2.2 Statistical Analysis

First, parametric and non‐parametric tests were used to examine the association between injury etiology and primary and secondary outcomes. Subsequently, a multivariate logistic regression model was used to evaluate whether injury etiology significantly predicted time to significant VSS improvement. Based on the multivariate logistic regression model, an interpretable prognostic nomogram was developed. The model calibration was assessed using calibration curves. Predictive performance was evaluated using receiver operating characteristic (ROC) curves and the corresponding area under the curve (AUC). Decision curve analysis (DCA) was conducted to quantify the clinical net benefit of the model. SHapley Additive exPlanations (SHAP) was used to enhance the interpretability of the predictive system and quantify the average contributions of features to identify key predictors of prognostic risk. Furthermore, it analyzed case‐specific decision pathways to clarify predictive outcomes for each individual patient, and this approach in turn boosted transparency in the model's reasoning process while reinforcing its clinical utility. Finally, the Chi‐square test was used to explore the correlations between injury etiology and demographic characteristics, injury severity, disease features, and treatment regimens.

### 2.3 Quantitative Statistical Analysis

All statistical analyses were performed with R version 4.4.3 (Institute for Statistics and Mathematics, Vienna, Austria) (https://www.r‐project.org). Mean ± standard deviation and median with range were used for the continuous variables, and count (percentage) was used for the categorical variables in descriptive statistics. Chi‐square tests were applied in the difference examination of categorical variables. Results with two‐sided *p* < 0.05 were regarded statistically significant.

## 3 Results

### 3.1 Demographic and Clinical Characteristics of Patients

**FIGURE 1:**
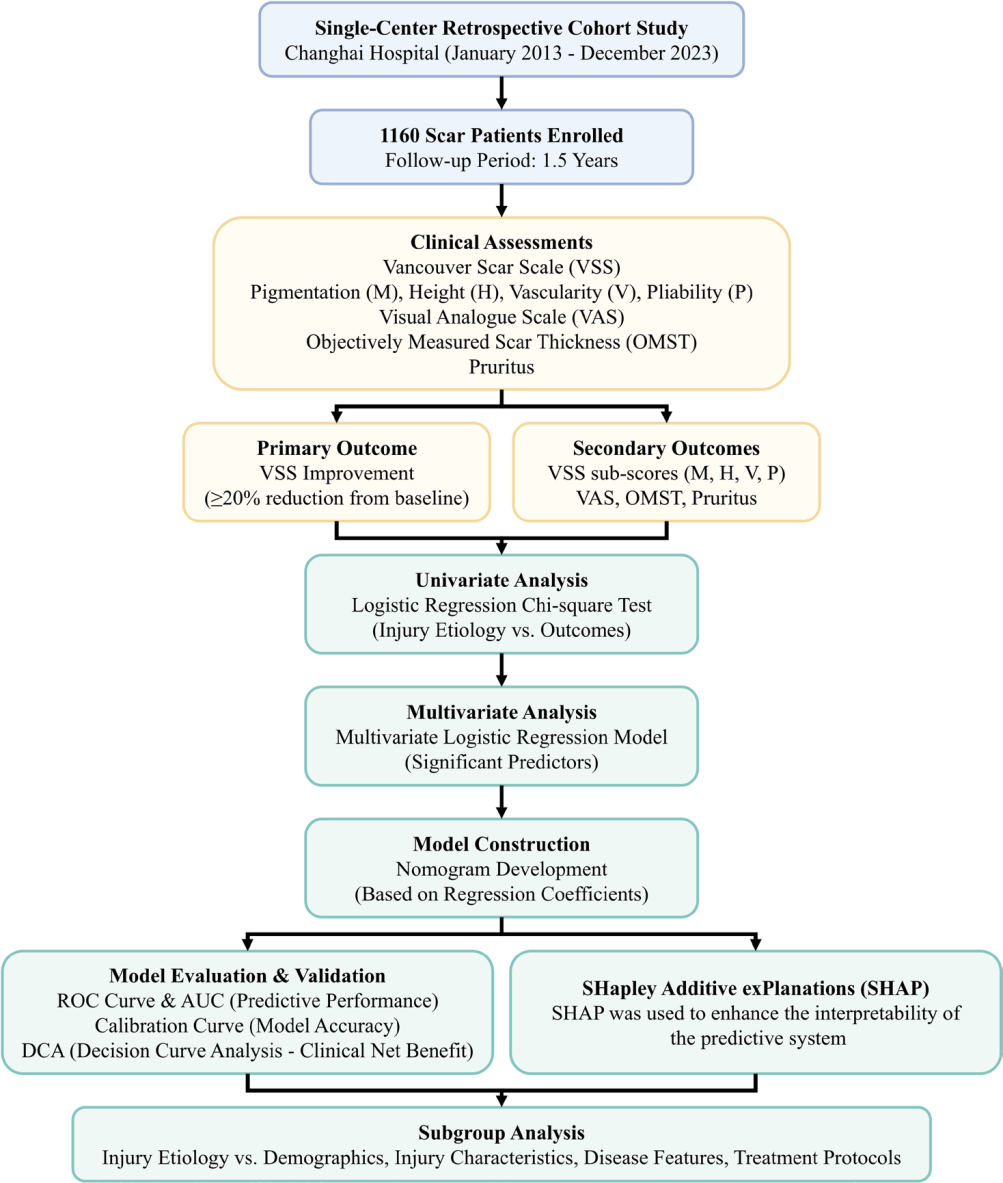
Overall study workflow. Flowchart of the single‐center retrospective cohort study including 1 160 scar patients with 1.5‐year follow‐up. Clinical assessments used the Vancouver Scar Scale (VSS), Visual Analogue Scale (VAS), objectively measured scar thickness (OMST), and pruritus evaluation. Analyses included univariate/multivariate regression, nomogram construction, model validation (ROC, calibration curve, DCA), SHAP interpretability analysis, and subgroup analysis. Abbreviations: ROC, receiver operating characteristic; DCA, decision curve analysis; SHAP, SHapley Additive exPlanations.

The overall analysis workflow of this study is illustrated in Figure 1. Detailed baseline characteristics of the patients are summarized in Table 1. A total of 1 160 patients were included, with a median age of 24 years (range, 0–78 years), and 50.95% were male. The majority (89.83%) resided in Eastern China. Regarding injury etiology distribution, burns were the predominant etiology (68.88%), followed by trauma (12.67%) and surgery (15.52%). For injury‐related features, full‐thickness skin injury accounted for 38.88%, while partial‐thickness injury accounted for 35.95%. Wound healing was ultimately achieved in 91.55% of patients, though 45.09% had a healing time exceeding 28 days. Manifest scars developed in 94.48% of patients, among whom 89.14% were diagnosed with hypertrophic scars. The majority of scars (54.66%) formed 29–60 days post‐injury. At the initial visit, 65.34% of scars were in the proliferative phase, and 21.55% were in the mature phase. The primary treatment goal was symptom control (69.83%). For the primary outcome, 33.88% of patients achieved significant VSS improvement, 44.40% showed partial improvement, and 18.62% showed no improvement. Among secondary outcomes, the pruritus relief rate was the highest (37.76%), followed by improvement in V (36.38%). Additional scar characteristics, treatment regimens, scar stage follow‐up data, and longitudinal treatment patterns are provided in Table S1.

**TABLE 1:** Descriptive statistics of demographic and clinical characteristics of patients.

Variables	Number (%)	Mean ± standard deviation	Median (range)
Gender			
Female	569 (49.05)		
Male	591 (50.95)		
Age		24.94 ± 19.48	24.00 (0.00–78.00)
< 19	469 (40.43)		
19–40	385 (33.19)		
41–60	228 (19.66)		
61–78	62 (5.34)		
Unknown	16 (1.38)		
Cause			
Burn	799 (68.88)		
Surgery	115 (9.91)		
Trauma	147 (12.67)		
Others	87 (7.50)		
Unknown	12 (1.03)		
Depth category			
Suture	180 (15.52)		
Full thickness	451 (38.88)		
Part‐thickness	417 (35.95)		
Unknown	112 (9.66)		
Wound healing time		43.83 ± 37.03	30.00 (5.00–360.00)
0–14d	195 (16.81)		
15–28d	234 (20.17)		
> 28d	523 (45.09)		
Unknown	208 (17.93)		
Scar classification			
Atrophic	72 (6.21)		
Hypertrophic	1 034 (89.14)		
Keloid	18 (1.55)		
Unknown	36 (3.10)		
Scar formation time		58.21 ± 109.72	40.00 (8.00–2555.00)
8–14d	42 (3.62)		
15–28d	102 (8.79)		
29–60d	634 (54.66)		
61–90d	96 (8.28)		
> 90d	65 (5.60)		
Unknown	221 (19.05)		
Treatment goal			
Appearance improvement	292 (25.17)		
Control of symptoms	810 (69.83)		
Function improvement	45 (3.88)		
Wound healing	12 (1.03)		
Unknown	1 (0.09)		
VSS improvement			
Non‐symptom	31 (2.67)		
Sig. improvement	393 (33.88)		
Non‐sig. improvement	338 (29.14)		
Unknown	398 (34.31)		

Abbreviation: VSS, Vancouver scar scale.

### 3.2 Burns Are Associated With Worse Scar Outcomes

**FIGURE 2:**
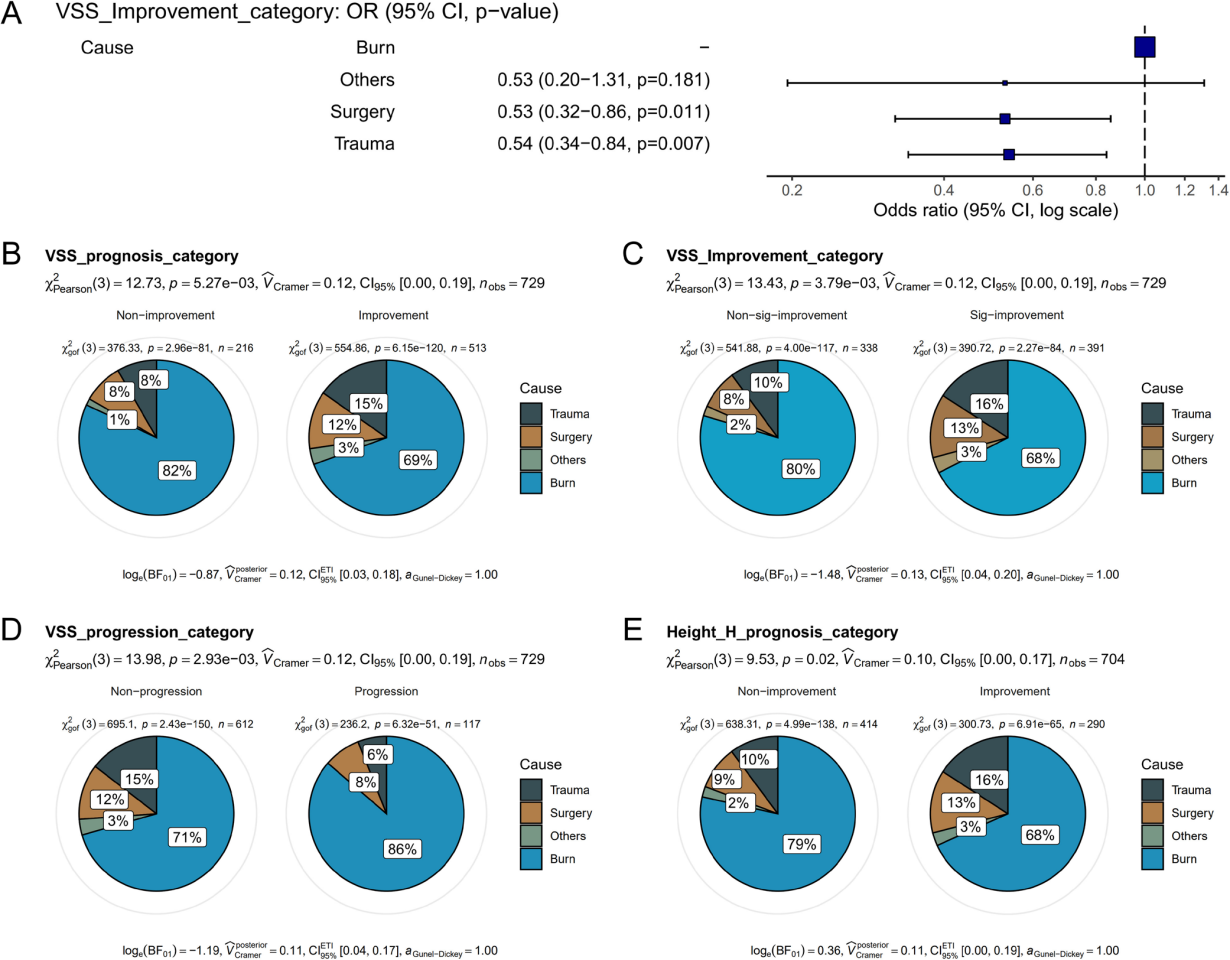
Injury etiology and scar outcome associations. (A) Forest plot of univariate odds ratios (ORs) for significant VSS improvement (burn as reference). (B–E) Pie charts showing etiology distribution across (B) overall VSS prognosis, (C) significant VSS improvement, (D) VSS progression, and (E) scar height prognosis.

Univariate logistic regression showed that burns were significantly associated with worse scar outcomes compared with surgery and trauma (Figure 2A, *p* < 0.05). Consistently, Chi‐square analysis showed that patients with burn‐related scars had the lowest overall improvement rates (Figure 2B–D, *p* < 0.05). In analyses of individual VSS components, burn‐related scars were associated with higher scar H scores (Figure 2E, Figure S1A, *p* < 0.05). Univariate regression suggested an association between surgical scars and M scores (Figure S1B, *p* < 0.05). However, this finding was not confirmed by Chi‐square analysis (Figure S1C, *p* > 0.05). Injury etiology was not significantly associated with V, P, VAS pain scores, pruritus, or OMST (Figure S1D–H, *p* > 0.05).

### 3.3 Injury Etiology Is a Significant Predictor of Scar Prognosis

**FIGURE 3:**
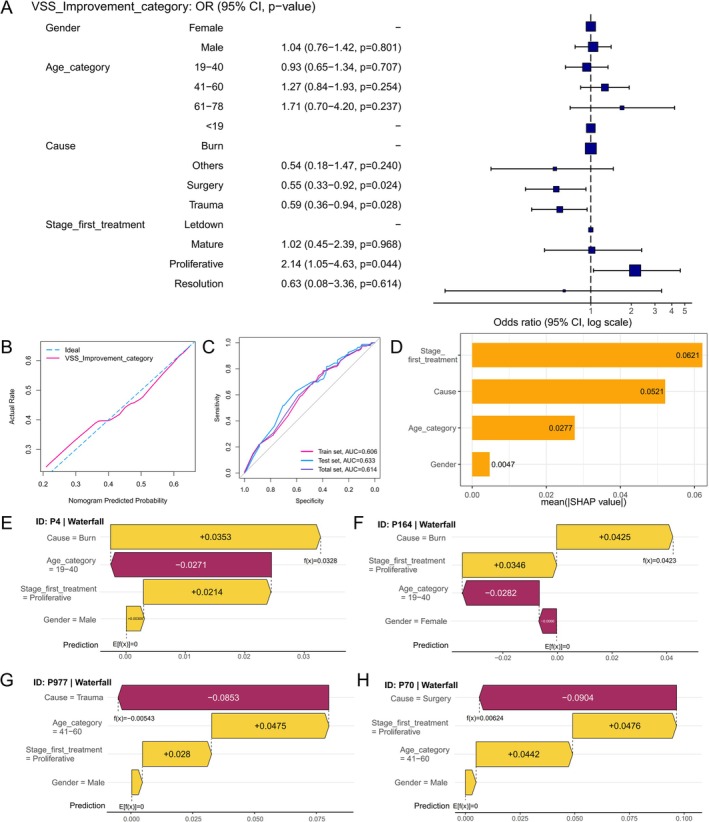
Prognostic model construction and validation. (A) Multivariate logistic regression forest plot for VSS improvement predictors. (B) Calibration curve of the nomogram. (C) ROC curves. (D) Mean SHAP values for feature importance. (E–H) SHAP waterfall plots for four representative patients.

Multivariate logistic regression analysis incorporating gender, age, scar stage at initial treatment, and injury etiology revealed that burns were a significant risk factor for impaired VSS improvement (Figure 3A, *p* < 0.05). Based on the multivariate logistic regression, a prognostic nomogram incorporating these variables was developed (Figure S2A and Table S2). In the scoring system, burns were assigned a risk score of 51 points, far higher than trauma (7 points), surgery (2 points), and other etiologies (0 points), indicating that burn patients had the lowest probability of achieving significant VSS improvement. Validation of the multivariate logistic regression prognostic model demonstrated good agreement between predicted and observed values via the calibration curve (Figure 3B). ROC curve analysis demonstrated modest discriminatory performance (Figure 3C, Train set AUC = 0.606, Test set AUC = 0.633, Total set AUC = 0.614). DCA suggested the clinical utility of the model in practical clinical settings (Figure S2B).

SHAP was used to quantify the contribution of features to scar prognosis risk prediction in the model, providing both global (population‐level) and local (individual‐level) insights. Global interpretation showed that the stage at the first treatment was the most influential predictor (SHAP value = 0.0621), followed by injury etiology (SHAP value = 0.0521) (Figure 3D, Figure **S2C**). The SHAP value scatter plot presented the impact of various categories of different clinical features on the model's prediction (Figure S2D–G). The stage at the first treatment and injury etiology were the main driving characteristics, with age having only a slight impact and gender having no substantial effect on prediction. SHAP visualization of typical cases embodied this mechanism. The SHAP waterfall plot demonstrated that injury etiology exerted an impact on the main orientation of the predicted values. Burns served as a positive driving cause, whereas trauma and surgery functioned as negative driving causes (Figure 3E–H). Burns represented a stable, positive contributing factor, but they remained susceptible to the influence of the treatment stage. The positive effect of burns underwent superimposition during the proliferative phase, while the negative contribution offset the positive effect of burns during the non‐proliferative phase (Figure S2H). Age also acted as an auxiliary factor, and the regulatory role of advanced age appeared more pronounced (Figure S2I).

### 3.4 Correlations Between Injury Etiology and Other Variables

Variables included in subgroup analyses and corresponding *p*‐values are shown in Table 2 and Table S3. Analysis of demographic characteristics revealed that burn patients were predominantly adolescents and children aged < 19 years, while surgical and traumatic patients were mainly young and middle‐aged adults aged 19–40 years. A higher proportion of females was observed in the surgical group (*p* < 0.05). Regarding injury and scar characteristics, significant differences in wound features were identified among different injury etiologies. Burn wounds were characterized by a larger area, greater depth, and longer healing time (*p* < 0.05). Burn‐related scars exhibited a longer formation latency and often involved multiple anatomical sites, particularly joints, hands, feet, upper/lower extremities, and the posterior trunk (*p* < 0.05). In contrast, surgical scars were mostly located on the anterior trunk (especially the anterior chest) (*p* < 0.05), while traumatic scars were more common on the knees, head, neck, and face (particularly the forehead) (*p* < 0.05). No significant differences in scar distribution were observed in other anatomical sites (*p* > 0.05).

**TABLE 2:** The variables and corresponding *p*‐values for the subgroup analyses.

Variables	Burn	Trauma	Surgery	Others	*p*
Gender	52: 48	49: 51	36: 64	63: 37	< 0.001*
Male; female					
Age	44: 30: 22: 4	35: 48: 10: 6	19: 45: 23: 12	59: 30: 8: 2	< 0.001*
< 19; 19–40; 41–60; 61–78					
TBSA	9: 23: 17: 30: 7: 6: 4: 4: 1	37: 35: 8: 6: 9: 3: 1: 1: 1	31: 39: 11: 9: 3: 2: 3: 2: 0	16: 23: 20: 39: 0: 0: 0: 0: 2	< 0.001*
≤ 0.1%; 0.11%–0.5%; 0.51%–1%; 1.1%–5%; 5.1%–10%; 11%–20%; 21%–30%; 31%–50%; 50%–100%					
Depth	54: 43: 3	14: 53: 33	4: 2: 94	10: 60: 30	< 0.001*
Full thickness; Part‐thickness; Suture					
Wound healing time	0: 6: 15: 22: 57	14: 25: 27: 17: 16	13: 75: 5: 4: 2	0: 20: 0: 20: 60	< 0.001*
0–7d; 8–14d; 15–21d; 22–30d; > 30d					
Scar formation time	2: 8: 34: 38: 11: 6: 1	10: 12: 45: 21: 7: 3: 2	14: 7: 42: 21: 8: 6: 3	0: 0: 43: 29: 14: 0: 14	< 0.001*
8–14d; 15–21d; 22–30d; 31–60d; 61–90d; 91–180d; > 180d					
Scar classification	98: 0: 1	93: 5: 2	96: 3: 2	24: 72: 4	< 0.001*
Hypertrophic; Atrophic; Keloid					
Multiple location	5	2	0	0	< 0.001*
Joint	24	27	14	4	< 0.001*
Treatment goal	75: 20: 5: 1	67: 31: 2: 1	74: 22: 4: 0	26: 73: 0: 1	< 0.001*
Control of symptoms; Appearance improvement; Function improvement; Wound healing					
Number of treatments	32: 23: 15: 31	33: 27: 14: 27	28: 26: 18: 28	41: 26: 16: 16	0.18
Once; Twice; Thrice; More than thrice					
Stage First treatment	74: 20: 5: 1	70: 21: 7: 2	76: 16: 6: 2	25: 72: 1:1	< 0.001*
Proliferative; Mature: Letdown; Resolution					
Stage 3 months after first treatment	65: 18: 12:5	61: 20: 17: 2	63: 9: 28: 0	30: 70: 0: 0	< 0.001*
Proliferative; Mature: Letdown; Resolution					
Stage 9 months after first treatment	30: 31: 27: 12	9: 59: 27: 5	56: 38: 6: 0	18: 73: 0: 9	0.005*
Proliferative; Mature: Letdown; Resolution					
Stage 12 months after first treatment	22: 46: 22: 10	6: 50: 38: 6	67: 20: 7: 7	0: 60: 20: 20	0.01*
Proliferative; Mature: Letdown; Resolution					

*Note:* Variables with *p* < 0.05 were signed with “*”.

Abbreviation: TBSA, total body surface area.

Notable differences in treatment strategies were observed across groups (*p* < 0.05). The primary treatment goal for burn patients was symptom control, with preferential use of combined regimens including topical medications (silicone‐based products, onion extract, asiaticoside), pressure therapy (elastic garments), and laser therapy, while injection therapy was less frequently utilized (*p* < 0.05). There was no significant difference in the number of treatment sessions among groups, with most patients receiving only one course of treatment (*p* > 0.05). Over time, the overall treatment compliance showed a downward trend. Follow‐up data indicated that burn and traumatic scars gradually transitioned from the proliferative phase to the mature phase over time, whereas surgical scars maintained a high proportion in the proliferative phase after treatment.

## 4 Discussion

Conventional scar assessment and prognosis have predominantly relied on phenotypic characteristics observed at end‐stage healing [12, 13]. In contrast, our study revealed a pivotal finding that injury etiology acted as a significant predictor of scar prognosis and served as an early risk signal available long before scar maturation, offering a critical opportunity for preemptive management. Importantly, injury etiology should not be interpreted in isolation, but rather as one component of a broader, interacting risk profile that includes healing duration, anatomical location, and patient‐specific factors. Burn injuries received a substantially higher risk score in the prognostic nomogram than trauma or surgical etiologies did. This finding indicates a stronger association between burn injuries and adverse outcomes. The specific correlation could be attributed to the unique pathophysiological characteristics of thermal injury. Unlike the controlled trauma of surgery, thermal energy induces widespread coagulative necrosis, triggering a sustained, potent immune response that favors hyperactivated fibroblasts and disordered collagen deposition [14, 15, 16, 17]. This mechanistic insult not only influences the biological trajectory of wound healing and fibrosis from the outset but also links to prolonged healing times, a known critical threshold for hypertrophic scarring risk [18, 19]. Our findings align with clinical consensus that etiology does not dictate fate but significantly alters scarring trajectories by modulating healing time, inflammatory intensity, and anatomical susceptibility, emphasizing its role in proactively flagging high‐risk patients rather than merely explaining outcomes retrospectively.

Our data support several practical escalation rules that burn injuries should be managed as high‐risk from the outset, surgical scars in high‐tension regions warrant intensified surveillance, and any wound requiring more than 21 days to heal should prompt escalation to a high‐risk scar management pathway, regardless of etiology. For burns, this high‐risk designation must be applied at the time of injury (not contingent upon later scar appearance), mandating immediate multimodal scar management upon re‐epithelialization rather than deferral until hypertrophy is evident. Pediatric burn patients face further amplified risk due to growth‐related skin tension [20, 21], necessitating a separately escalated management protocol. While surgically closed wounds typically heal rapidly by primary intention (confining the inflammatory phase and yielding more favorable prognoses), surgery is not universally low‐risk in that its risk is highly anatomy‐dependent. Specifically, anterior chest scars (a high‐tension region) exhibit a persistent proliferative phase, a finding that highlights the need for high‐risk protocols for surgical scars in such zones (e.g., anterior chest, shoulders, back), and intraoperative tension‐reduction techniques are as critical as postoperative interventions in these regions with persistent postoperative proliferation signaling the need for therapy escalation. For traumatic scars, the risk should be dynamically assessed based on the healing course on the principle that any wound, regardless of etiology, that requires more than 21 days to heal merits automatic high‐risk management, and traumatic wounds complicated by repeated debridement, infection, or severe crush injury, which impose healing challenges and inflammatory burdens similar to those of burns, should be managed with burn‐level vigilance.

Our findings exposed a critical care gap that, despite bearing the highest risk profile, burn patients in the present cohort experienced delayed and inconsistent prophylactic therapy, with low initiation of multimodal treatment within the crucial first month post‐healing. This discrepancy underscores that current workflows fail to translate etiological risk stratification into timely action, compounded by the profound psychosocial burden of burns that may hinder compliance [22]. Thus, scar management must evolve from a reactive phenotype‐driven model to a proactive etiology‐guided strategy that implements default early intervention for burns, anatomically stratified protocols for surgical scars, and healing time‐based risk escalation for all wounds.

This study has several limitations. First, there is currently no universally accepted, standardized threshold for defining clinically significant improvement on the VSS in the international scar research community. Our decision to use a 20% reduction in VSS score as the cutoff for clinically meaningful relief was made to balance minimizing false‐positive results from random scoring fluctuations and avoiding false‐negative results that would miss clinically relevant improvements. To validate the robustness of our findings, we performed sensitivity analyses using four additional threshold gradients, namely 10%, 20%, 30%, and 50% (Figure S3). The rationale for selecting 20% over alternative thresholds is as follows. A 10% threshold was deemed excessively low, as VSS scores are susceptible to minor random variations from inter‐rater differences, diurnal fluctuations in scar appearance, and subtle changes in lighting conditions during assessment, which would substantially increase the risk of false‐positive classification of relief. Conversely, thresholds of 30% or higher were considered overly stringent, as they would exclude a significant proportion of patients who experienced clinically observable and meaningful improvements in scar symptoms (such as reduced pruritus, pain, and erythema) despite not achieving a 30% reduction in VSS score. Second, the analysis was based solely on objective clinician‐assessed VSS scores and did not incorporate validated patient‐reported outcome measures. The correlation between specific VSS reduction thresholds and patient‐perceived meaningful improvement in scar symptoms remains to be established in future prospective studies. Third, as a single‐center retrospective study, it is susceptible to selection bias, and the results need to be validated in multi‐center cohorts with diverse ethnic and geographic backgrounds. Fourth, the “trauma” group included heterogeneous subtypes such as lacerations, crush injuries, and avulsion injuries, which may have different scarring trajectories and warrant further investigation in future studies with finer classification. Fifth, a pervasive methodological limitation of observational scar research is that observed clinical improvements may simply reflect the natural maturation trajectory of proliferative scars rather than genuine therapeutic effects. This study explicitly addresses this critical concern by clarifying its research design, objectives, and findings as follows. This study was designed as a retrospective cohort study assessing scar outcomes under standard clinical practice, not a randomized controlled trial (RCT) intended to establish causal efficacy of any individual therapeutic intervention. The primary objective was to identify early prognostic factors, particularly injury etiology, for clinical risk stratification, rather than to quantify the absolute treatment effect of specific interventions. To mitigate confounding by the natural scar maturation process, this study rigorously adjusted for “stage at first treatment” as an independent variable in the multivariate logistic regression model. SHAP analysis further confirmed that this variable was the most impactful predictor of scar outcomes (SHAP value = 0.0621), exceeding even injury etiology in its predictive strength. This finding demonstrates that the observed association between injury etiology and scar prognosis is independent of baseline scar maturation stage, thereby eliminating the potential bias that proliferative scars might exhibit higher apparent improvement rates solely due to their natural evolutionary course. Furthermore, stratified analysis demonstrated that even within scars in the identical proliferative phase, burn‐induced scars exhibited a significantly lower improvement rate compared with both surgical and traumatic scars. This result provides additional confirmatory evidence that injury etiology represents an independent determinant of scar prognosis, distinct from the effects of natural maturation.

## 5 Conclusion

This study investigated the association between injury etiology and scar prognosis and indicated that burn injury was a significant predictor of worse scar outcomes. This high risk can be attributed to various factors related to thermal injury, including its propensity to cause deep tissue damage, trigger a sustained inflammation, and result in prolonged wound healing times. It can be inferred that injury etiology serves as a crucial early warning signal for scar risk, underscoring the importance of prioritizing proactive, etiology‐based risk stratification over reactive management based on scar phenotype. As a result, future scar management strategies should prioritize early, etiology‐based risk stratification. Early identification of high‐risk patients, particularly those with burns, may support timely and appropriate clinical management to optimize scar outcomes.

## Author Contributions

R.H., L.Z., and M.M. contributed to conceptualization, data curation, formal analysis, investigation, visualization, and writing – original draft. D.X., H.B., and S.J. contributed to project administration, funding acquisition, supervision, and writing – review and editing. The authors declare that they have no known competing financial interests or personal relationships that could have appeared to influence the work reported in this paper. All authors have read and approved the final manuscript.

## Funding

This study was jointly supported by National Key R&D Program of China (2024YFA1108405); National Natural Science Foundation of China (82472546); Science and Technology Innovation Project of Shanghai Science and Technology Committee (25CL2900703); Shanghai Top Priority Research Center Project (2023ZZ02013); Excellent Academic Leader Project of Shanghai Science and Technology Committee (23XD1425000); Deep Blue Talent Project of Naval Medical University; The Sanhang Talent Program of Naval Medical University; The ChangFeng Talent Development Program of The First Affiliated Hospital of Naval Medical University; Postdoctoral Fellowship Program of CPSF (GZC20242278); Shanghai Rising‐Star Program (Sailing Special Program) (No. 23YF1458400). The funders had no role in study design, data collection and analysis, decision to publish, or preparation of the manuscript.

## Ethics Statement

The study was approved by the Ethics Committee of the First Affiliated Hospital of Naval Medical University (CHEC2024‐410).

## Conflicts of Interest

The authors declare no conflicts of interest.

## Supporting information


**Figure S1:** Etiology associations with secondary scar outcomes.
(A and B) Forest plots of univariate ORs for VSS height and pigmentation improvement (burn as reference). (C–H) Pie charts showing etiology distribution across improvement subgroups for VSS sub scores, VAS pain, pruritus, and OMST.
**Figure S2:** Supplementary nomogram validation and SHAP analysis.
(A) Prognostic nomogram for VSS non‐improvement probability. (B) Decision curve analysis of clinical utility. (C) SHAP beeswarm plot of global feature impact. (D–G) SHAP dependence plots for individual predictors. (H and I) SHAP waterfall plots for two additional representative patients.
**Figure S3:** Sensitivity analysis with varying VSS improvement thresholds.
Forest plots showing multivariate regression results using four outcome definitions: (A) 10%, (B) 20% (primary threshold), (C) 30%, and (D) 50% VSS score reduction.
**Table S1:** On clinical characteristics of patients.
**Table S2:** Nomogram points for these four variables.
**Table S3:** The variables and corresponding *p*‐values for the subgroup analyses.

## Data Availability

The data that support the findings of this study are available on request from the corresponding author. The data are not publicly available due to privacy or ethical restrictions.
