# Supplementary material for: Association Between Injury Etiology and Scar Outcomes: A Retrospective Clinical Study
Source: J Cosmet Dermatol. 2026 Jul 9;25(7):e71042. doi: 10.1111/jocd.71042 (PMC13351825; doi:10.1111/jocd.71042)
Supplement: Supplementary file 1 — Figure S1: Etiology associations with secondary scar outcomes. (A and B) Forest plots of univariate ORs for VSS height and pigmentation improvement (burn as reference). (C–H) Pie charts showing etiology distribution across improvement subgroups for VSS sub scores, VAS pain, pruritus, and OMST. Figure S2: Supplementary nomogram validation and SHAP analysis. (A) Prognostic nomogram for VSS non‐improvement probability. (B) Decision curve analysis of clinical utility. (C) SHAP beeswarm plot of global feature impact. (D–G) SHAP dependence plots for individual predictors. (H and I) SHAP waterfall plots for two additional representative patients. Figure S3: Sensitivity analysis with varying VSS improvement thresholds. Forest plots showing multivariate regression results using four outcome definitions: (A) 10%, (B) 20% (primary threshold), (C) 30%, and (D) 50% VSS score reduction. Table S1: On clinical characteristics of patients. Table S2: Nomogram points for these four variables. Table S3: The variables and corresponding p‐values for the subgroup analyses. [file JOCD-25-e71042-s001.docx]

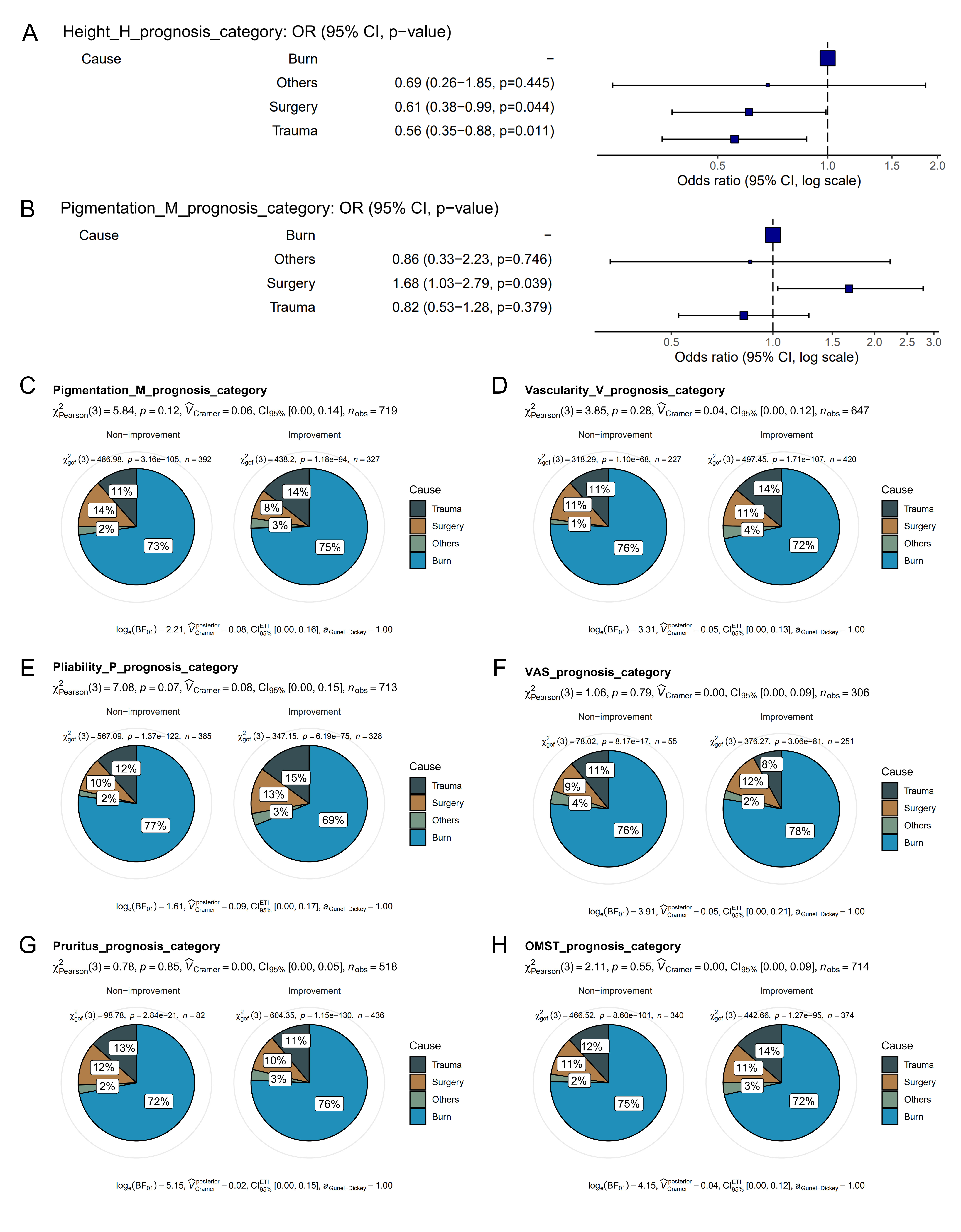
 **Figure S1. Etiology associations with secondary scar outcomes**

(A and B) Forest plots of univariate ORs for VSS height and pigmentation improvement (burn as reference). (C-H) Pie charts showing etiology distribution across improvement subgroups for VSS sub scores, VAS pain, pruritus, and OMST.

**
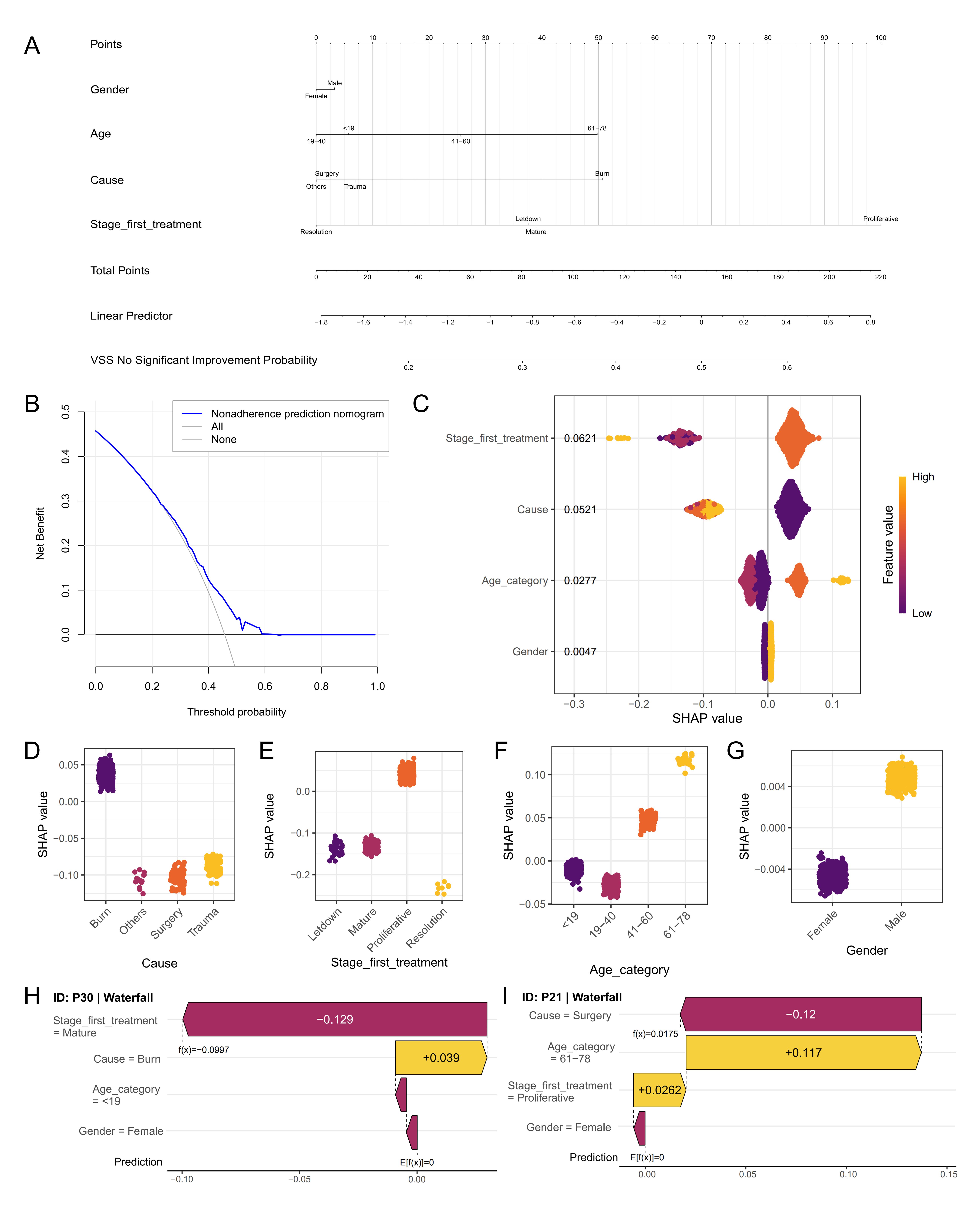
**

**Figure S2. Supplementary nomogram validation and SHAP analysis**

(A) Prognostic nomogram for VSS non-improvement probability. (B) Decision curve analysis of clinical utility. (C) SHAP beeswarm plot of global feature impact. (D-G) SHAP dependence plots for individual predictors. (H and I) SHAP waterfall plots for two additional representative patients.

**
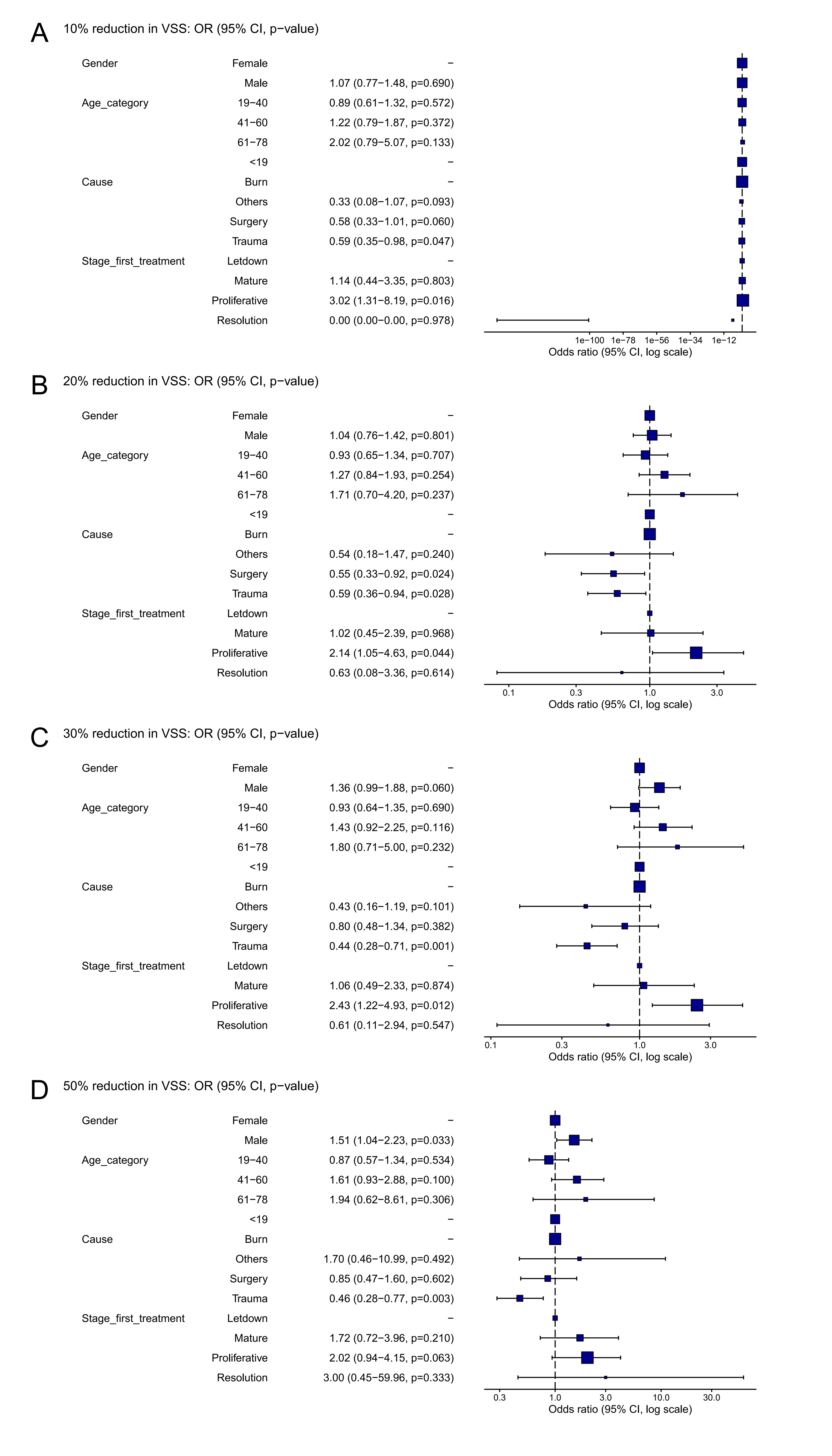
**

**Figure S3. Sensitivity analysis with varying VSS improvement thresholds**

Forest plots showing multivariate regression results using four outcome definitions: (A) 10%, (B) 20% (primary threshold), (C) 30%, and (D) 50% VSS score reduction.

**Table S1** Supplementary data on clinical characteristics of patients

| **Variables** | **Number (%)** | **Mean ± standard**  **deviation** | **Median (range)** |
| --- | --- | --- | --- |
| **Residence category** |  |  |  |
| Central South China | 58 (5.00) |  |  |
| East China | 1,042 (89.83) |  |  |
| Foreign countries | 3 (0.26) |  |  |
| North China | 9 (0.78) |  |  |
| Northeast China | 8 (0.69) |  |  |
| Northwest China | 8 (0.69) |  |  |
| Southwest China | 22 (1.90) |  |  |
| Unknown | 10 (0.86) |  |  |
| **Wound healing** |  |  |  |
| Yes | 1,062 (91.55) |  |  |
| No | 49 (4.22) |  |  |
| Unknown | 49 (4.22) |  |  |
| **TBSA** |  | 0.05 ± 0.11 | 0.01 (0.00-1.00) |
| ≤ 0.1% | 166 (14.31) |  |  |
| 0.11%-0.5% | 281 (24.22) |  |  |
| 0.51%-1% | 165 (14.22) |  |  |
| 1.1%-5% | 274 (23.62) |  |  |
| 5.1%-10% | 67 (5.78) |  |  |
| 11%-20% | 51 (4.40) |  |  |
| 20%-30% | 41 (3.53) |  |  |
| 31%-50% | 31 (2.67) |  |  |
| 50%-100% | 11 (0.95) |  |  |
| Unknown | 73 (6.29) |  |  |
| **Joint** |  |  |  |
| Yes | 174 (15.00) |  |  |
| No | 641 (55.26) |  |  |
| Unknown | 345 (29.74) |  |  |
| **Multiple location** |  |  |  |
| Yes | 44 (3.79) |  |  |
| No | 1,104 (95.17) |  |  |
| Unknown | 12 (1.03) |  |  |
| **Pigmentation prognosis** |  |  |  |
| Non-symptom | 41 (3.53) |  |  |
| Improvement | 329 (28.36) |  |  |
| Non-improvement | 392 (33.79) |  |  |
| Unknown | 398 (34.31) |  |  |
| **Vascularity prognosis** |  |  |  |
| Non-symptom | 113 (9.74) |  |  |
| Improvement | 422 (36.38) |  |  |
| Non-improvement | 227 (19.57) |  |  |
| Unknown | 398 (34.31) |  |  |
| **Height prognosis** |  |  |  |
| Non-symptom | 56 (4.83) |  |  |
| Improvement | 292 (25.17) |  |  |
| Non-improvement | 414 (35.69) |  |  |
| Unknown | 398 (34.31) |  |  |
| **Pliability prognosis** |  |  |  |
| Non-symptom | 47 (4.05) |  |  |
| Improvement | 330 (28.45) |  |  |
| Non-improvement | 385 (33.19) |  |  |
| Unknown | 398 (34.31) |  |  |
| **VAS prognosis** |  |  |  |
| Non-symptom | 402 (34.66) |  |  |
| Improvement | 253 (21.81) |  |  |
| Non-improvement | 55 (4.74) |  |  |
| Unknown | 450 (38.79) |  |  |
| **Pruritus prognosis** |  |  |  |
| Non-symptom | 191 (16.47) |  |  |
| Improvement | 438 (37.76) |  |  |
| Non-improvement | 82 (7.07) |  |  |
| Unknown | 449 (38.71) |  |  |
| **OMST prognosis** |  |  |  |
| Non-symptom | 17 (1.47) |  |  |
| Improvement | 376 (32.41) |  |  |
| Non-improvement | 340 (29.31) |  |  |
| Unknown | 427 (36.81) |  |  |
| **Stage at the first treatment** |  |  |  |
| Proliferative | 758 (65.34) |  |  |
| Mature | 250 (21.55) |  |  |
| Letdown | 59 (5.09) |  |  |
| Resolution | 15 (1.29) |  |  |
| Unknown | 78 (6.72) |  |  |
| **Stage 1 month after the first treatment** | | | |
| Proliferative | 366 (31.55) |  |  |
| Mature | 37 (3.19) |  |  |
| Letdown | 25 (2.16) |  |  |
| Resolution | 5 (0.43) |  |  |
| Unknown | 727 (62.67) |  |  |
| **Stage 3 months after the first treatment** | | | |
| Proliferative | 283 (24.40) |  |  |
| Mature | 92 (7.93) |  |  |
| Letdown | 62 (5.34) |  |  |
| Resolution | 17 (1.47) |  |  |
| Unknown | 706 (60.86) |  |  |
| **Stage 6 months after the first treatment** | | | |
| Proliferative | 121 (10.43) |  |  |
| Mature | 91 (7.84) |  |  |
| Letdown | 54 (4.66) |  |  |
| Resolution | 21 (1.81) |  |  |
| Unknown | 873 (75.26) |  |  |
| **Stage 9 months after the first treatment** | | | |
| Proliferative | 47 (4.05) |  |  |
| Mature | 63 (5.43) |  |  |
| Letdown | 38 (3.28) |  |  |
| Resolution | 15 (1.29) |  |  |
| Unknown | 997 (85.95) |  |  |
| **Stage 12 months after the first treatment** | | | |
| Proliferative | 34 (2.93) |  |  |
| Mature | 63 (5.43) |  |  |
| Letdown | 31 (2.67) |  |  |
| Resolution | 14 (1.21) |  |  |
| Unknown | 1,018 (87.76) |  |  |
| **Stage 1.5 years after first treatment** |  |  |  |
| Proliferative | 10 (0.86) |  |  |
| Mature | 62 (5.34) |  |  |
| Letdown | 15 (1.29) |  |  |
| Resolution | 6 (0.52) |  |  |
| Unknown | 1,067 (91.98) |  |  |
| **Treatment 1 month after wound healing** | | | |
| Yes | 392 (33.79) |  |  |
| No | 662 (57.07) |  |  |
| Unknown | 106 (9.14) |  |  |
| **First treatment day after injury** |  | 848.46 ± 1,782.01 | 169.00 (4.00-14,192.00) |
| < 30 | 79 (6.81) |  |  |
| 30-60 | 137 (11.81) |  |  |
| 61-90 | 105 (9.05) |  |  |
| 91-180 | 207 (17.84) |  |  |
| 181-365 | 157 (13.53) |  |  |
| 366-730 | 111 (9.57) |  |  |
| > 730 | 226 (19.48) |  |  |
| Unknown | 138 (11.90) |  |  |
| **First treatment after injury in 90 days** | | | |
| Yes | 321 (27.67) |  |  |
| No | 701 (60.43) |  |  |
| Unknown | 138 (11.90) |  |  |
| **Number of treatments** |  | 2.96 ± 2.37 | 2.00 (1.00-17.00) |
| Once | 378 (32.59) |  |  |
| Twice | 273 (23.53) |  |  |
| Thrice | 173(14.91) |  |  |
| More than thrice | 336 (28.97) |  |  |
| **Laser** |  |  |  |
| Yes | 1,126 (97.07) |  |  |
| No | 29 (2.50) |  |  |
| Unknown | 5 (0.43) |  |  |
| **Fractional laser at the first treatment** | | | |
| Yes | 909 (78.36) |  |  |
| No | 245 (21.12) |  |  |
| Unknown | 6 (0.52) |  |  |
| **IPL at the first treatment** |  |  |  |
| Yes | 238 (20.52) |  |  |
| No | 916 (78.97) |  |  |
| Unknown | 6 (0.52) |  |  |
| **Surgical treatment at the first treatment** | | | |
| Yes | 44 (3.79) |  |  |
| No | 1,110 (95.69) |  |  |
| Unknown | 6 (0.52) |  |  |
| **Injection treatment at the first treatment** | | | |
| Yes | 19 (1.64) |  |  |
| No | 1,135 (97.84) |  |  |
| Unknown | 6 (0.52) |  |  |
| **Topical drugs** |  |  |  |
| Yes | 578 (49.83) |  |  |
| No | 518 (44.66) |  |  |
| Unknown | 64 (5.52) |  |  |
| **Injection** |  |  |  |
| Yes | 39 (3.36) |  |  |
| No | 1,057 (91.12) |  |  |
| Unknown | 64 (5.52) |  |  |
| **Elastic clothing** |  |  |  |
| Yes | 146 (12.59) |  |  |
| No | 950 (81.90) |  |  |
| Unknown | 64 (5.52) |  |  |
| **Anesthesia method** |  |  |  |
| General anesthesia | 175 (15.09) |  |  |
| Local anesthesia | 652 (56.21) |  |  |
| Nerve blocking anesthesia | 12 (1.03) |  |  |
| No | 82 (7.07) |  |  |
| Unknown | 239 (20.60) |  |  |
| **Flushing drug** |  |  |  |
| Yes | 894 (77.07) |  |  |
| No | 171 (14.74) |  |  |
| Unknown | 95 (8.19) |  |  |
| **Dressing** |  |  |  |
| Yes | 40 (3.45) |  |  |
| No | 1,014 (87.41) |  |  |
| Unknown | 106 (9.14) |  |  |
| **Glucocorticoid** |  |  |  |
| Yes | 497 (42.84) |  |  |
| No | 567 (48.88) |  |  |
| Unknown | 96 (8.28) |  |  |
| **Silicone** |  |  |  |
| Yes | 385 (33.19) |  |  |
| No | 775 (66.81) |  |  |
| **Onion extract** |  |  |  |
| Yes | 149 (12.84) |  |  |
| No | 1,011 (87.16) |  |  |
| **Asiaticoside** |  |  |  |
| Yes | 135 (11.64) |  |  |
| No | 1,025 (88.36) |  |  |
| **Treatment 1 month after the first treatment** | | | |
| Yes | 452 (38.97) |  |  |
| No | 698 (60.17) |  |  |
| Unknown | 10 (0.86) |  |  |
| **Treatment 3 months after the first treatment** | | | |
| Yes | 478 (41.21) |  |  |
| No | 675 (58.19) |  |  |
| Unknown | 7 (0.60) |  |  |
| **Treatment 6 months after the first treatment** | | | |
| Yes | 294 (25.34) |  |  |
| No | 854 (73.62) |  |  |
| Unknown | 12 (1.03) |  |  |
| **Treatment 9 months after the first treatment** | | | |
| Yes | 165 (14.22) |  |  |
| No | 982 (84.66) |  |  |
| Unknown | 13 (1.12) |  |  |
| **Treatment 12 months after the first treatment** | | | |
| Yes | 148 (12.76) |  |  |
| No | 995 (85.78) |  |  |
| Unknown | 17 (1.47) |  |  |
| **Treatment 1.5 years after the first treatment** | | | |
| Yes | 95 (8.19) |  |  |
| No | 1,050 (90.52) |  |  |
| Unknown | 15 (1.29) |  |  |
| **Highest energy at the first treatment** |  | 55.80 ± 44.28 | 35.00 (2.50-150.00) |
| 2.5-25 | 266 (22.93) |  |  |
| 26-50 | 198 (17.07) |  |  |
| 51-100 | 196 (16.90) |  |  |
| 101-150 | 124 (10.69) |  |  |
| Unknown | 376 (32.41) |  |  |
| **Highest density at the first treatment** |  | 4.34 ± 1.13 | 5.00 (1.00-5.00) |
| 1-3% | 213 (18.36) |  |  |
| 4-5% | 552 (47.59) |  |  |
| Unknown | 395 (34.05) |  |  |
| **Highest frequency at the first treatment** | | 243.98 ± 33.18 | 250.00 (100.00-400.00) |
| < 250 | 37 (3.19) |  |  |
| 250-400 | 657 (56.64) |  |  |
| Unknown | 466 (40.17) |  |  |
| **Unit at the first treatment** |  | 21.87 ± 40.92 | 10.00 (1.00-400.00) |
| <= 10 | 456 (39.31) |  |  |
| 11-49 | 238 (20.52) |  |  |
| >= 50 | 79 (6.81) |  |  |
| Unknown | 387 (33.36) |  |  |
| Abbreviations: TBSA, total body surface area; VAS, visual analog scale; OMST, objective measured scar thickness; IPL, intense pulsed light. | | | |

**Table S2** Nomogram points for these four variables

| **Variables** | **Points** |
| --- | --- |
| **Gender** |  |
| Female | 0 |
| Male | 3 |
| **Age** |  |
| < 19 | 6 |
| 19-40 | 0 |
| 41-60 | 26 |
| 61-78 | 50 |
| **Cause** |  |
| Burn | 51 |
| Surgery | 2 |
| Trauma | 7 |
| Others | 0 |
| **Stage at the first treatment** |  |
| Proliferative | 100 |
| Mature | 39 |
| Letdown | 38 |
| Resolution | 0 |
| **Total Points VSS No Significant Improvement Probability** |  |
| 184 | 0.6 |
| 150 | 0.5 |
| 117 | 0.4 |
| 80 | 0.3 |
| 36 | 0.2 |

Abbreviations: VSS, Vancouver scar scale.

**Table S3** The variables and corresponding p values for the subgroup analyses

| **Variables** | **Burn** | **Trauma** | **Surgery** | **Others** | **p value** |
| --- | --- | --- | --- | --- | --- |
| **Residence** | 89: 6: 6 | 95: 3: 2 | 96: 2: 3 | 95: 4: 1 | 0.57 |
| East; Central South; Others |  |  |  |  |  |
| **Hand** | 19 | 20 | 9 | 1 | < 0.001* |
| **Foot** | 9 | 5 | 4 | 1 | 0.02* |
| **Upper limb** | 36 | 25 | 24 | 5 | < 0.001* |
| **Lower limb** | 30 | 28 | 11 | 1 | < 0.001* |
| **Back trunk** | 6 | 1 | 1 | 1 | 0.006* |
| **Front trunk** | 15 | 1 | 38 | 14 | < 0.001* |
| **Front chest** | 11 | 1 | 20 | 13 | < 0.001* |
| **Knee** | 1 | 9 | 2 | 0 | < 0.001* |
| **Head face and neck** | 39 | 45 | 30 | 78 | < 0.001* |
| **Forehead** | 2 | 9 | 2 | 5 | < 0.001* |
| **Shoulder** | 2 | 0 | 3 | 2 | 0.3 |
| **Elbow** | 1 | 3 | 1 | 0 | 0.07 |
| **Wrist** | 1 | 1 | 4 | 0 | 0.09 |
| **Hip** | 3 | 0 | 1 | 0 | 0.07 |
| **Ankle** | 1 | 0 | 0 | 0 | 0.36 |
| **Back** | 4 | 1 | 1 | 2 | 0.11 |
| **Topical drugs** | 61 | 42 | 43 | 8 | < 0.001* |
| **Elastic clothing** | 18 | 4 | 6 | 0 | < 0.001* |
| **Laser** | 98 | 98 | 93 | 99 | 0.01* |
| **Injection** | 2 | 4 | 8 | 8 | < 0.001* |
| **Silicone** | 38 | 27 | 31 | 1 | < 0.001* |
| **Onion extract** | 16 | 8 | 7 | 3 | < 0.001* |
| **Asiaticoside** | 15 | 4 | 6 | 1 | < 0.001* |
| **Stage 1 month after first treatment** | 85: 9: 5: 1 | 80: 7: 11: 2 | 87: 6: 4: 2 | 82: 18: 0: 0 | 0.7 |
| Proliferative; Mature: Letdown; Resolution |  |  |  |  |  |
| **Stage 6 months after first treatment** | 43: 32: 18: 7 | 32: 39: 23: 6 | 50: 8: 33: 8 | 36: 57: 7: 0 | 0.12 |
| Proliferative; Mature: Letdown; Resolution |  |  |  |  |  |
| **Stage 1.5 years after first treatment** | 9: 67: 18: 6 | 8: 83: 8: 0 | 33: 33: 22: 11 | 0: 80: 0: 20 | 0.25 |
| Proliferative; Mature: Letdown; Resolution |  |  |  |  |  |
| **Treatment 1 month after wound healing** | 40 | 36 | 30 | 11 | < 0.001* |
| **Treatment 1 month after first treatment** | 42 | 41 | 43 | 14 | < 0.001* |
| **Treatment 3 months after first treatment** | 43 | 40 | 41 | 35 | 0.46 |
| **Treatment 6 months after first treatment** | 28 | 23 | 20 | 20 | 0.13 |
| **Treatment 9 months after first treatment** | 14 | 15 | 14 | 14 | 0.99 |
| **Treatment 12 months after first treatment** | 14 | 12 | 14 | 9 | 0.62 |
| **Treatment 1.5 years after first treatment** | 9 | 8 | 8 | 8 | 0.98 |

Variables with p < 0.05 were signed with “*”.
